# The main component of the aphid alarm pheromone (*E*)-β-farnesene affects the growth and development of *Spodoptera exigua* by mediating juvenile hormone-related genes

**DOI:** 10.3389/fpls.2022.863626

**Published:** 2022-08-23

**Authors:** Yang Sun, Yan Li, Wen Zhang, Bin Jiang, Si-Min Tao, Han-Yang Dai, Xin-Tong Xu, Yue-Xin Sun, Lei Yang, Yong-Jun Zhang

**Affiliations:** ^1^Key Laboratory for Conservation and Use of Important Biological Resources of Anhui Province, Anhui Provincial Key Laboratory of Molecular Enzymology and Mechanism of Major Diseases, College of Life Sciences, Anhui Normal University, Wuhu, China; ^2^Institute of Plant Protection, Jiangsu Academy of Agricultural Sciences, Nanjing, China; ^3^Laboratory for Biology of Plant Diseases and Insect Pests, Institute of Plant Protection, Chinese Academy of Agricultural Sciences, Beijing, China

**Keywords:** *Spodoptera exigua*, (E)-β-farnesene, RNA-seq, *SeVg* and *SeVgR*, reproduction and oviposition

## Abstract

The (*E*)-β-farnesene (EβF) is one of the most important secondary metabolites in some plants and provides indirect defense against aphids. However, the direct effect of EβF against pests is still unclear. In this study, various concentrations of EβF (0.16, 0.8, and 4 g/kg) were provided in an artificial diet to determine the direct effects of EβF on *Spodoptera exigua*. The results showed that an artificial diet containing 4 g/kg of EβF reduced the final survival of the *S. exigua* larvae and per female fecundity of adults significantly when compared with CK and SC controls (*p* < 0.05), then ultimately it also significantly affected the intrinsic rate of increase (*p* < 0.05). Furthermore, the results of the EβF bioassay in an artificial diet also indicated that the proliferation of the *S. exigua* population was inhibited by the ingestion of EβF in a dose-dependent manner. Combined differential RNA-seq data and RT-qPCR analysis, it was found that four key genes involved in juvenile hormone degradation significantly upregulated in *S. exigua* larvae treated by EβF at a dose of 0.8 and 4 g/kg when compared with two controls (*p* < 0.05). This indicated that EβF could disturb the normal function of juvenile hormones and reduce the survival rate of *S. exigua* larvae. Additionally, two key genes that regulate per fecundity of *S. exigua* females, including *SeVg* and *SeVgR*, were significantly downregulated in adult females (*p* < 0.05) when they were treated with 0.8 and 4 g/kg of EβF at the larval stage, relative to the expression of these genes after treatment with controls. These findings suggested that EβF first disturbed the normal function of juvenile hormone by upregulating key degradation genes, and then inhibited the expression of *SeVg*/*SeVgR* genes and proteins, thus reducing the population size of *S. exigua* by increasing larval mortality and inhibiting per female fecundity.

## Introduction

*Spodoptera exigua* is a major polyphagous pest that affects crops, such as alfalfa, soybean, peanut, onion, corn, scallion, and cotton, leading to huge economic losses (Saeed et al., 2010; Zhang et al., 2011; Hafeez et al., 2020). *S. exigua* is mainly controlled by spraying chemical pesticides (Saeed et al., 2019; Huang et al., 2021). However, the development of insecticide resistance (one of the “3R” major concerns) occurs due to the long-term and improper use of pesticides on agricultural products (Zhen and Gao, 2016). To prevent the target insects from developing resistance to insecticides, novel bioactive molecules with high efficiency but low toxicity to non-target organisms are required in agricultural production (Zhen and Gao, 2016).

Plants have co-evolved with insects and produce various secondary metabolites to defend against pests (Hu et al., 2021). The secondary metabolites from plants can be divided into four categories: Terpenoids, phenols, alkaloids, and sulfur-containing compounds. These metabolites exhibit toxicity, antifeedant activity, and antibiosis effects on pests (Adeyemi, 2010; War et al., 2012). Among these phytochemicals, terpenoids are the most structurally diverse (Nagegowda and Gupta, 2020). Terpenoids are synthesized from the five-carbon isopentenyl diphosphate (IPP) and isomer dimethylallyl diphosphate (DMAPP) (Sun et al., 2017; Michael et al., 2020; Zhou and Pichersky, 2020). With the advancement in molecular biology, many key enzymes that are required for the synthesis of terpenes [e.g., terpene synthases (TPSs)] have been identified in *Arabidopsis thaliana* (Chen et al., 2004).

Recently, many TPS coding genes were found in gramineous crops, such as *TPS*10 in corn, *TPS*46, and *TPS*23 in rice. These genes play a key role in indirect defense against insect pests (Yuan et al., 2008; Capra et al., 2015; Sun et al., 2017). Among the products of these three TPS genes, the products of *TPS*10 and *TPS*46 have very similar amino acid sequences (Sun et al., 2017). Interestingly, both *TPS*10 and *TPS*46 synthesize the same volatile substance (E)-β-farnesene (EβF) (Schnee et al., 2006; Yuan et al., 2008; Sun et al., 2017).

The compound EβF plays an important role in crop pest control, especially in controlling aphids (Beale et al., 2006; Cui et al., 2012). Overexpression of *tps*10 in *A. thaliana* not only repelled aphids but also attracted aphid parasitoids, whereas the overexpression of *tps*46 in rice also repelled bird cherry-oat aphid (Beale et al., 2006; Sun et al., 2017). The latest research showed that EβF had presented a magic attractive effect on the larvae and adults of the aphid predator hoverfly *Eupeodes corollae* (Wang et al., 2022). Additionally, EβF was also used to attract the natural enemies of cabbage aphids to control these pests in Chinese cabbage fields (Cui et al., 2012; Su et al., 2015). With the advancement in molecular biology, the TPSs of EβF have also been identified in many other plants, such as *Matricaria recutita* (L.), *Douglas-fir*, and *Mentha piperita* (Huber et al., 2005; Yu et al., 2013; Su et al., 2015).

Terpenes from many plants have been reported to be toxic to pests. Four monoterpenes, including β-phellandrene, α-pinene, p-paracymene, and (+)-2-mainly carene, produced in greenhouse tomato flowers, showed direct toxicity to insects (Morse et al., 2012). Previous studies also showed that EβF, as one of the important products of *TPS*46, has a direct lethal effect on many other phytophagous insects (Cui et al., 2012). Therefore, EβF and the TPSs associated with it might be applied as substitutes for chemical pesticides to protect plants. In our previous study, EβF showed a strong lethal effect on the larvae of the *Chilo suppressalis*, and consequently, *C. suppressalis* failed to complete its life cycle (Yang et al., 2022). However, *S. exigua* could complete its life cycle when treated with the same concentration of EβF as used for controlling *C. suppressalis*. This indicated that there was a difference in the response of *S. exigua* and *C. suppressalis* to EβF. Therefore, to determine the mechanism by which EβF affects the life cycle of insects, especially that of adults. We used differential RNA-seq, Western blotting, and bioinformatics techniques to systematically analyze the life table data and the expression of key genes in *S. exigua* after treatment with different sublethal doses of EβF. Therefore, the physiological and molecular responses of *S. exigua* after being treated with EβF were elucidated, which might provide a basis for determining the natural compounds to control *S. exigua* in crops.

## Materials and methods

### Rearing of *Spodoptera exigua*

The initial colony of *S. exigua* was provided by the Institute of Plant Protection, Jiangsu Academy of Agricultural Sciences, and the larvae were raised on an artificial diet following an established method (Ren et al., 2013) in the laboratory without exposure to pesticides at 27 ± 1°C, relative humidity of 65 ± 5% under a 14:10 light/dark cycle. The adults of *S. exigua* were provided with 10% honey solution as food in a previous study (Zhao et al., 2018).

### Preparing different concentrations of (*E*)-β-farnesene for *Spodoptera exigua* treatments

The EβF was added to the artificial diet of the larvae (Yang et al., 2022). Based on our previous study (Sun et al., 2017), the five concentration gradients of EβF (18794–84–8, Sigma-Aldrich), including 0.032, 0.16, 0.8, 4, and 20 g/kg (EβF/artificial diet, standard compound of EβF/uncured liquid feed), were set for initial testing. In the pre-experiment phase, treatment of *S. exigua* larvae with 0.032 g/kg of EβF showed no noticeable effect, while treatment with 20 g/kg of EβF killed all the larvae. Meanwhile, the standard EβF purchased from Sigma-Aldrich was very expensive, and the use of 20 g/kg of EβF/artificial diet required huge consumption. Additionally, the mortality of *S. exigua* larvae treated with 4 g/kg of EβF/artificial diet was close to 50%, which was equivalent to the lethal medium concentration (*LC*_50_). Therefore, 4 g/kg of EβF/artificial diet was set as the maximum treatment dose by considering the cost. Next, three concentration gradients of EβF were selected (0.16, 0.8, and 4 g/kg), as well as ethanol and water treatments were used as solvent control (SC) and blank control (CK), respectively. The newly hatched first instar larvae of *S. exigua* were exposed to five treatments, including 0.16, 0.8, 4 g/kg, SC, and CK, and their entire life cycle was monitored for recording specific effects during different stages of development. In the larval stage, the mixed artificial diet was constantly changed to maintain the dosage stability of EβF on the larvae. The *S. exigua* larvae were placed in a small perforated plastic box (10 cm × 6 cm × 5 cm) and fed with an artificial diet. Each treatment had five replicates, and each replicate had 200 larvae, which were maintained in 40 small plastic boxes (five larvae in each box).

### Sample collection and construction of the life table

Following previously described methods (Sun et al., 2016; Zhao et al., 2018), the life table of *S. exigua* was constructed and included data on the mortality of the larvae at different instars, per female fecundity, the hatching rate, the intrinsic rate of increase, and other key parameters (Table 1). The parameters were recorded for each treatment from multiple insects maintained in separate boxes. The data collected from multiple insects were summarized to construct the life table.

**TABLE 1:** The key life-history parameters of *S. exigua* larvae under different doses of EβF.

Key life history parameters of *S. exigua*	Different treatments				
	
	Controls		Different doses of Eβ F		
		
	CK	SC	0.16 g/kg	0.8 g/kg	4 g/kg
Total mortality of larvae at 1st and 2nd instars (%)	4.38 ± 0.87c	3.33 ± 1.36c	5.00 ± 1.71c	11.67 ± 4.38b	28.33 ± 6.18a
Larvae mortality of 3rd instar (%)	1.46 ± 0.93a	1.04 ± 0.74a	1.67 ± 1.58a	2.08 ± 1.65a	2.71 ± 0.94a
Larvae mortality of 4th instar (%)	1.88 ± 1.36a	2.29 ± 1.36a	1.87 ± 0.87a	1.46 ± 1.19a	2.50 ± 1.19a
Ultimate larvae mortality (%)	14.17 ± 1.58c	17.50 ± 2.00c	19.17 ± 2.72c	36.67 ± 5.02b	54.75 ± 7.10a
Larval stage (d)	12.64 ± 1.64a	12.78 ± 1.94a	13.13 ± 1.92a	13.55 ± 2.52a	14.12 ± 3.08a
The whole life-span of *S. exigua* (d)	31.19 ± 3.02a	31.37 ± 3.04a	31.65 ± 3.37a	32.79 ± 4.30a	34.43 ± 6.04a
Adult emergence rate (%)	78.25 ± 9.98a	79.11 ± 7.93a	74.66 ± 8.71a	72.70 ± 8.77a	70.52 ± 8.15a
Per female fecundity	512.40 ± 57.67a	504.80 ± 45.86a	464.20 ± 62.01a	370.00 ± 54.19b	155.40 ± 32.11c
Hatching rate of eggs (%)	68.47 ± 7.14a	67.11 ± 6.92a	66.60 ± 7.68a	63.38 ± 6.79a	57.98 ± 6.89a
Intrinsic rate of increase (R)	0.154 ± 0.018a	0.151 ± 0.012a	0.144 ± 0.017a	0.122 ± 0.019b	0.079 ± 0.012c

CK (blank control) and SC (solvent control) were set as controls. The concentrations of EβF treatments were set as 0.16, 0.8, and 4 g/kg; the units of g/kg in all treatment groups mean the mass ratio of EβF/artificial diet. Data are presented by mean ± standard error; different lower-case letters indicate a significant difference obtained by ANOVA, followed by Tukey’s HSD test (p < 0.05).

Adult *S. exigua* were placed in a lidless plastic box (20 cm × 20 cm × 20 cm) with the top covered with gauze to lay eggs in the dark. Before hatching, the eggs were transferred to sterile glass jars and administered an artificial diet. They were incubated under normal conditions (27 ± 1°C; 65 ± 5% RH; 14:10 light/dark cycle). To determine the hatching rate, five groups of egg clothes were randomly selected to count the number of eggs in a clutch under a microscope, and then the number of hatched larvae was counted to evaluate the hatching rate. To determine per female fecundity, 10 pairs of *S. exigua* adults were selected from each treatment group, and the experiment was repeated five times. The per female fecundity was evaluated by calculating the average number of eggs laid for each pair of male and female adults.

During the experiment, *S. exigua* was sampled twice; the initial sampling was performed at the beginning of the third instar of *S. exigua*, and the final sampling was performed when the adult females were 2 days old. The initial sampling was performed at the beginning of the third instar because the first and second instar EβF-treated larvae had high mortality, but the third and fourth instar larvae had almost no deaths. We sampled 50 third instar larvae of *S. exigua* for each treatment, and five repeats were performed. Half of the samples were used for performing differential RNA-seq to detect the changes in the key genes in the surviving *S. exigua* larvae, while the other half of the samples were used for performing real-time quantitative PCR (RT-qPCR).

The second sampling was performed when the adult females were 2 days old to measure the gene and protein expressions of SeVg and SeVgR. Additionally, the fat body and tissue samples of the ovary from these adult females treated with different doses of EβF were dissected and collected. Preliminary experiments showed that EβF treatment strongly inhibited per female fecundity of *S. exigua*. Our previous study also showed that SeVg and SeVgR are the key proteins for evaluating the reproductive potential of adult females and their expression in female *S. exigua* can reach the maximum level within 2 days (Sun et al., 2016; Zhao et al., 2018). Therefore, 10 2-day-old female adults were taken from each treatment and repeated the experiment three times. Thus, a total of 30 females were used to detect the expression of SeVg and SeVgR proteins. Additionally, two 2-day-old adult females were taken for each treatment, repeated five times, and a total of 20 females were used to detect the expression of *SeVg* and *SeVgR* genes by performing RT-qPCR.

### RNA isolation, library preparation, and PacBio sequencing

Total RNA has extracted from *S. exigua* larvae at the beginning of the third instar using the TRIzol reagent (Invitrogen, Carlsbad, CA, United States) strictly as directed by the manufacturer. The integrity of the RNA was determined with the Agilent 2100 Bioanalyzer (Agilent Technologies, Palo Alto, California, United States). Total RNA samples with RIN value ≥ 8 were used for constructing the cDNA libraries in PacBio sequencing. Using the Clontech SMARTer PCR cDNA Synthesis Kit (Takara Biotechnology, Dalian, China), 4 μg RNA is synthesized to cDNA and subsequently amplified to generate double-stranded cDNA. The cDNA was then the size selected for < 4 kb and > 4 kb fractions using the BluePippin Size Selection System (Sage Science, Beverly, MA, United States). Each SMRTbell library was constructed using 1 μg size-selected cDNA with the Pacific Biosciences SMRTbell template prep kit. The binding of SMRT bell templates to polymerases was conducted using the Sequel II Binding Kit, and then primer annealing was performed. Sequencing was carried out on the Pacific Bioscience Sequel II platform.

For short-read sequencing, magnetic beads with Oligo (dT) were used to enrich eukaryotic mRNAs for constructing the sequencing library. Next, a fragmentation buffer was added to break the mRNAs into short fragments. Then, the RNA-seq libraries were constructed using the NEBNext^®^ Ultra™ RNA Library Prep Kit for Illumina^®^ (NEB, United States), following the manufacturer’s instructions. The different libraries were pooled for sequencing on the Illumina HiSeq platform. Sequencing of all samples was conducted by Nanjing Genepioneer Biotechnologies Inc. (Nanjing, China).

### Unigene assembly and annotation

The standard protocol of ISO-seq (SMRT Analysis 2.3) was followed to process the raw PacBio full-length ISO-seq data. The reads of inserts (ROIs) were obtained from the circular consensus sequences (CCS). After searching for 5’ and 3’ adaptors and poly(A) signals, full-length, and non-full-length cDNA reads were defined, and chimeric reads were removed, including the sequencing primers. The redundant sequences were moved using CD-HIT-EST to obtain non-redundant (NR) high-quality transcripts (Fu et al., 2012). The sequences of these NR transcripts were defined as unigenes.

To determine the open reading frames (ORFs) present in the transcripts, we used TransDecoder v2.01^1^ to confirm the putative coding sequences (CDSs). The predicted CDSs were searched and confirmed by BLASTX (*E*-value ≤ 1e-5) against three protein databases [NR, SwissProt, and Kyoto Encyclopedia of Genes and Genomes (KEGG)]. The transcripts containing complete ORFs, 5’-untranslated regions (UTRs), and 3’-UTRs were regarded as FL transcripts. For further studying the juvenile hormone-related genes, we selected the genes related to juvenile hormone based on the information on the annotation of unigenes.

### Analysis of differentially expressed genes

Fastp v0.20.1 (Chen et al., 2018) was used to remove the adapters and low-quality sequences in the raw data. The short reads of each sample were aligned to the unigenes using Bowtie2 (Langmead et al., 2009). The read counts of the unigenes were obtained using htseq-count (Anders et al., 2015). The FPKM (Fragments Per Kilobase of transcript per Million mapped reads) of the unigenes in all samples were calculated as the expression level. RSEM was used to calculate the value of the FPKM (Li and Dewey, 2011). DESeq2 was used for differential expression analysis, and the unigenes with a cut-off of | log2 ratio| ≥ 1 and *q* < 0.05 were selected for significant differential expression (Love et al., 2014).

The FPKM values of the differentially expressed juvenile hormone-related genes were converted to log2 (FPKM) values. Then, the log2 (FPKM) values were used to plot the heatmap by ComplexHeatmap (Gu et al., 2016).

### RT-qPCR analysis

Total RNA of the *S. exigua* samples exposed to different treatments (CK, SC, and different doses of EβF) was extracted using the TRIzol reagent (Invitrogen, Carlsbad, CA, United States) strictly as directed by the manufacturer. An MMLV Reverse Transcriptase (Promega, Madison, WI, United States) treated with ribonuclease H (Takara, Tokyo, Japan) was used to synthesize the cDNA library, and spectrophotometry was performed to quantify the library. The primers used for RT-qPCR (SYBR Green I) were designed based on the genes that were found to be related to the juvenile hormone from the RNA-seq data (Table 2). Also, the house-keeping gene of *S. exigua*, β*-actin* was used as an endogenous reference for data normalization (Zhao et al., 2018). The primers of the house-keeping gene and the other 10 random gene sequences that were used to verify the results of differential RNA-seq are listed in Table 2. And the details of these 10 selected genes are listed in Supplementary Table 1.

**TABLE 2:** Sequences of RT-qPCR primers used in this study.

Gene	Upstream primer(5′-3′)	Downstream primer (5′-3′)	Tm (°C)	Product size (bp)
*SeVg*	5′-GCATACCAGCCAACTACCAAAT-3′	5′-TGCACCTGACACTGTCTACCCT-3′	60°C	149
*SeVgR*	5′-GAAGGGAGGGAAGTGTCCTGAG-3′	5′-TGATGGTGAAAGAAACGCTGTG-3′	60°C	104
*Actin*	5′-CCAGCCTTCCTTCTTGGGTAT-3′	5′-AGGTCCTTACGGATGTCAACG-3′	60°C	94
Unigene026066	5′-AGTTTGATGAAGCGATGA-3′	5′-TTAGTCTTGAAATGAGGGA-3′	50°C	199
Unigene025818	5′-ATCCCAACCACAGCACCAG-3′	5′-AATGCCCTCCATCATCCAG-3′	58°C	127
Unigene025841	5′-GCAATGGGTTTGGCTACT-3′	5′-TTTGTTGGCTGGGTCTGT-3′	54°C	232
Unigene024849	5′-TATGGGTTTATGTGCTTAGA-3′	5′-GTAGAACTGATGTGGCTCC-3′	50°C	163
Unigene022385	5′-GTATTAGCGAGGGAAACA-3′	5′-ACCAATCTGAGCAGCACT-3′	53°C	201
Unigene029506	5′-TGATGATGCTGGCTGAGAT-3′	5′-GAGGCTGGTGAAACAACTG-3′	60°C	286
Unigene029881	5′-CCCCGTCAGCAAGGTGG-3′	5′-CGTTGGCGTTGAATCCG-3′	60°C	214
Unigene029782	5′-GCCCACCCCAAATACGA-3′	5′-GTCAGCCTCCACCAGCG-3′	57°C	123
Unigene024091	5′-GTTGATGGGTGATTTGG-3′	5′-TACACTGGGTTGGTCGT-3′	50°C	105
Unigene024366	5′-GGAGTGTAAACAGAAGGGAGTC-3′	5′-GCTGGTCGCTGATGAAGA-3′	54°C	162

The Primer 5.0 software was used to design all EST-specific primers. The reactions were performed using the SYBR Premix Ex Taq Kit (Takara, Tokyo, Japan) and were conducted on the Bio-Rad iCycler real-time quantitative RT-PCR detection system. The RT-qPCR was conducted using a 25 μL reaction mixture, which contained 0.5 μL of each primer (10 μM) (total 1.0 μL), 12.5 μL of 2 × SYBR Premix Ex Taq, 2.0 μL of sample cDNA (100 ng), 0.5 μL of ROX Reference DYE, and sterilized H_2_O 9.0 μL. And the RT-qPCR parameters were set as follows: 5 min at 94°C; (35 cycles) 10 s at 94°C, 20 s at 53–58°C, and 15 s at 75°C. The melting curve analysis and gel electrophoresis were also performed to confirm the RT-qPCR quality of these genes. The reactions of each treatment were replicated four times; non-template control reactions were performed in triplicate for each primer pair. The relative expression levels of each gene in *S. exigua* exposed to different treatments (expressed as the relative quantification (RQ) values) were calculated using the 2^–ΔΔ^ *^Ct^* method (Livak and Schmittgen, 2001). Significant differences in the expression of the genes were determined by performing a one-way analysis of variance, followed by Duncan’s multiple comparison test.

### The relative expression *SeVg and SeVgR* in *Spodoptera exigua* treated with different doses of (*E*)-β-farnesene

*SeVg* and *SeVgR* were cloned in a previous study (Zhao et al., 2018). The specimens were snap-frozen in liquid nitrogen and kept at –80°C until used for total RNA extraction and RT-qPCR. Each treatment group had five biological replicates, with two adult females per replicate. The primers used for performing RT-qPCR of the *SeVg* and *SeVgR* genes were based on a previous study (Zhao et al., 2018), and are listed in Table 2.

### The relative expression of *SeVg and SeVgR* proteins in female adults treated with different doses of (*E*)-β-farnesene

The fat body and ovary tissue samples of more than 10 females were used to perform the Western blot analysis (Sun et al., 2016; Zhao et al., 2018). Total proteins were extracted using the Tissue Protein Extraction Reagent kit (Zoonbio Biotechnology Co., Ltd, Nanjing, China), and the concentrations of the proteins were determined by the bicinchoninic acid (BCA) method. The Western blot analysis was performed following the methods reported in a study by Song et al. (2012), with some modifications using the grayscale value of target protein/grayscale value of β-actin protein. Uniform spotted wells were used, the concentration of *S. exigua* protein in each well was equal (1 μg/μL), and 20 μL of the sample was loaded to ensure that each well contained about 20 μg. For each Western blot analysis, samples containing an equal amount of total proteins were resolved on the same gel. Protein samples were electrophoresed on 10% SDS-PAGE and electroblotted onto an NC membrane (Bio-Rad) running at 100 mA for 3 h with Tris/glycine buffer. The membranes were then blocked for 1 h at 37°C with 5% non-fat powdered milk in Tris-buffered saline containing 0.05% Tween 20 (TBS-T).

The primary antibody was specific and produced by the Zoonbio Biotechnology Co., Ltd, Nanjing, China. It is based on the SeVg and SeVgR gene and protein sequence. The 697 amino acids (aa) specific antibody sequence of SeVg was designed from 29 to 725 aa sequence of SeVg full-length protein (1,761 aa, accession number AOH73254.1 at the National Center for Biotechnology Information, NCBI), which contained 2,091 base pairs (bp) designing from 85 to 2,175 bp in the SeVg mRNA complete CDS (5,286 bp, accession number KT599434.1 in NCBI). The 256 aa specific antibody sequence of SeVgR was designed from 1,063 to 1,318 aa sequence of SeVg full-length protein (1,814 aa, accession number AOX13593.1 in NCBI), which contained 768 bp designed from 3,187 to 3,954 bp in the SeVgR mRNA complete CDS (5,445 bp, accession number KT899978.1 in NCBI). Then, the primary antibody was incubated overnight with the sample. The secondary antibody was a goat anti-rabbit antibody procured from the Boster Biological Technology (Catalog no. BA1054), which was incubated with the sample for 1–2 h.

### Statistical analysis

Statistical analyses were conducted using the SAS v.9.0 software (SAS Institute, Cary, NJ, United States). The data were presented as the mean ± standard deviation and analyzed by performing a one-way analysis of variance (ANOVA), followed by Tukey’s honest significant difference (HSD) test. According to Greenberg et al. (2001); Sun et al. (2016), and Zhao et al. (2018), the relationships between the key life-history parameters of *S. exigua* and the relative expression of SeVg or SeVgR genes were evaluated by linear regression analyses.

## Results

### (*E*)-β-farnesene effects on *Spodoptera exigua* at different concentration treatments

*S. exigua* treated with EβF/artificial diet (including 0.16, 0.8, and 4 g/kg) showed lower food intake, higher developmental disorder, higher mortality of the first and second instar larvae, and lower per female fecundity than that treated with CK and SC controls. The effects on the key life-history parameters of *S. exigua* larvae treated with different lethal doses of EβF are shown in Table 1. Non-significant differences were observed in per female fecundity, hatching rate of eggs, and intrinsic rate of increase between *S. exigua* individuals treated with 0.16 g/kg of EβF and those in the two controls (*p* > 0.05; Table 1). Nevertheless, compared with the CK and SC controls, the *S. exigua* larvae treated with EβF at the concentrations of 0.8 g/kg and 4 g/kg had a significant increase in the mortality rate of first and second instar larvae (*p* < 0.05; Table 1). The mortality rate of first and second instar larvae treated with 0.8 g/kg EβF was 11.67%, and with 4 g/kg could reach 28.33%. No significant difference was seen in the mortality of the third and fourth instar larvae treated with EβF at the doses of 0.8 and 4 g/kg in comparison with two controls (*p* > 0.05; Table 1). Significant differences in ultimate mortality of *S. exigua* larvae treated with EβF at the concentration of 0.8 and 4 g/kg (36.67 and 54.75%, respectively) were observed (*p* < 0.05; Table 1). These results showed that larval mortality was mostly affected by EβF in a dose-dependent manner, especially at 4 g/kg (*p* < 0.05; Table 1).

Compared with CK and SC controls, there were no significant differences in life-span, adult emergence rate, and hatching rate of eggs between 0.8 and 4 g/kg EβF treatments (*p* > 0.05; Table 1). However, two parameters of population build-up of *S. exigua*, i.e., per female fecundity and intrinsic rate of increase, had significant differences after treatment with 0.8 and 4 g/kg of EβF (*p* < 0.05; Table 1). The EβF concentration of 4 g/kg had the strongest effect on per female fecundity and the intrinsic rate of increase (R) in *S. exigua* larvae (*p* < 0.05; Table 1). These results indicated that the effect of EβF on *S. exigua* was stronger at higher doses.

Significant differences in larval survival and adult oviposition of *S. exigua* were found after treatment with 0.8 g/kg EβF (*p* < 0.05; Table 1) compared to the control, and the differences were more prominent at 4 g/kg of EβF (*p* < 0.05; Table 1). Thus, 0.8 and 4 g/kg of EβF effectively inhibited the growth of the *S. exigua* population (*p* < 0.05; Table 1).

### Functional annotation and differential expression analysis of unigenes

**FIGURE 1:**
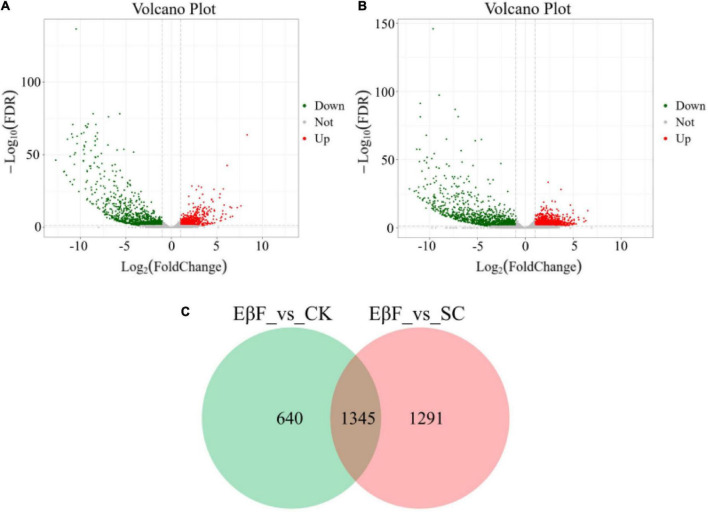
The mixed graphs showed the relative expression level of all differentially expressed unigenes in *S. exigua* larvae after EβF treatments at doses of 4 g/kg. **(A)** The volcano plot obtained from the DESeq2 analysis between 4 g/kg EβF treatments and CK (blank control) samples showing log_2_ (fold-change) and-Log_10_ FDR values. **(B)** The volcano plot obtained from DESeq2 analysis between 4 g/kg EβF treatments and SC (solvent control) samples showing log_2_ (foldchange) and-Log_10_ FDR values. **(C)** The numbers of DEGs among different treatments among CK, SC, and 4 g/kg EβF treatment. The concentrations of EβF treatments were set as 4 g/kg; the unit of EβF treatment as g/kg means the mass ratio of EβF/artificial diet. The value denotes the value of log_2_ (FPKM + 1).

The Pacbio sequencing platform generated 34,339 unigenes with an N50 value of 4,625 bp. A total of 30,009 unigenes were annotated in at least one public database, such as NR, NT, COG, GO, KEGG, Swissport, and Interpro protein databases. Finally, 17,473 unigenes were found to be expressed in at least one sample. Differential gene expression analysis was conducted between the samples in the EβF treatments and controls (SC and CK) with fold change levels ≥ 2 with an FDR ≤ 0.05. It resulted in the identification of 1,985 different expressed genes between EβF treatment and CK (Figure 1A). Of these, 1,161 unigenes were downregulated, while 824 genes were upregulated under EβF treatments. Meanwhile, 2,636 differentially expressed genes (DEGs) were identified between EβF treatments and SC (Figure 1B). In these DEGs, 1,372 were downregulated DEGs, and 1,264 were upregulated DEGs. Among these DEGs, 1,345 unigenes were differentially expressed both in the 4 g/kg vs. CK and 4 g/kg vs. SC (Figure 1C and Supplementary Tables 2, 3).

**FIGURE 2:**
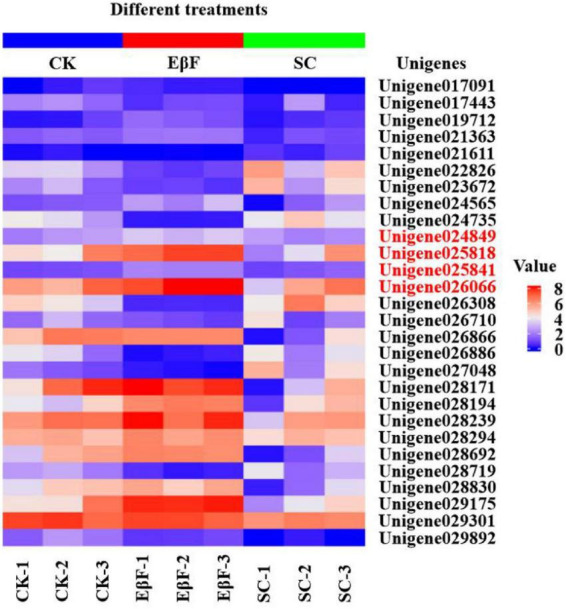
The heat map showed the relative expression level of all differentially expressed juvenile hormone-related unigenes in *S. exigua* larvae after EβF treatments at doses of 4 g/kg. CK (blank control) and SC (solvent control) were set as controls. The concentrations of EβF treatments were set as 4 g/kg; the units of EβF treatment as g/kg in all treatment groups mean the mass ratio of EβF/artificial diet. The value denotes the value of log2 (FPKM + 1). The unigenes for further study using the RT-qPCR were marked with red font.

Based on the annotation information, 48 juvenile hormone-related unigenes were identified. Eighteen juvenile hormone-related genes were differentially expressed between EβF treatment and CK. Additionally, 25 juvenile hormone-related genes were differentially expressed between the EβF treatment and the SC control (Figure 2). Among these differentially expressed juvenile hormone-related unigenes, 15 were differentially expressed in EβF treatment vs. CK and EβF treatment vs. SC. In total, 28 juvenile hormone-related genes were differentially expressed (Figure 2).

**FIGURE 3:**
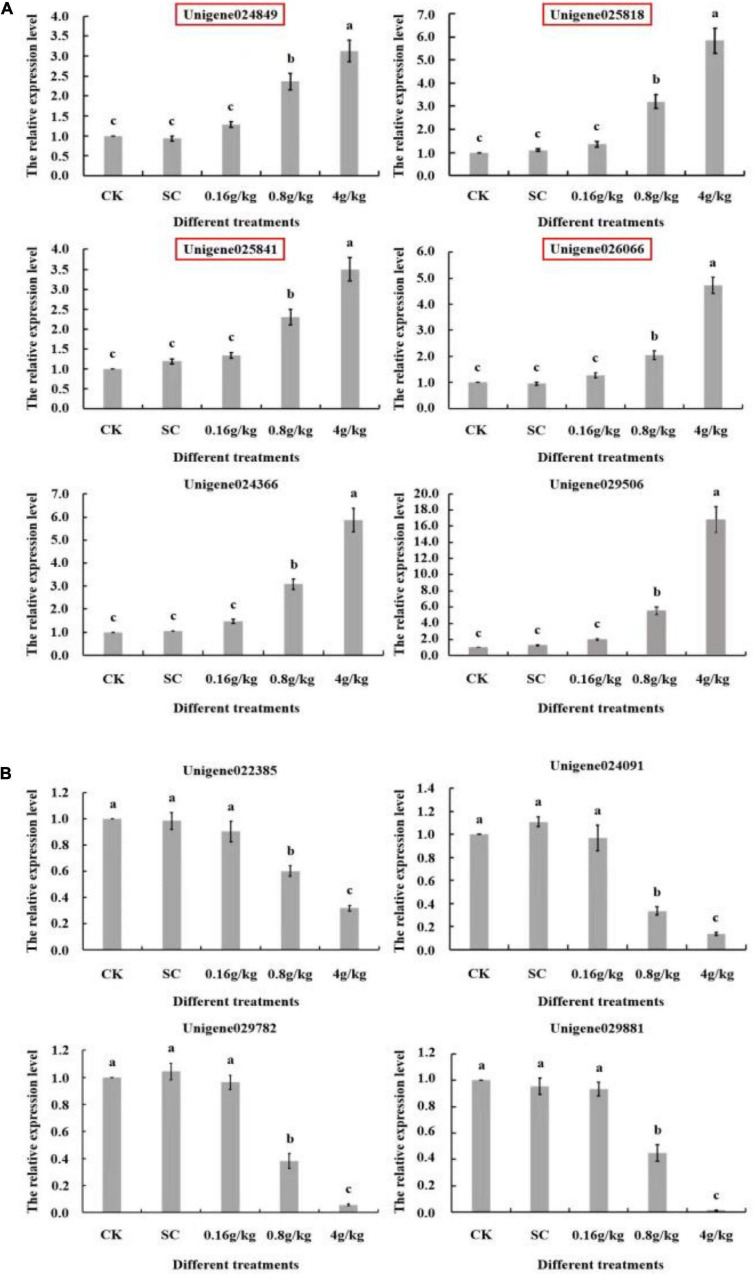
The relative expression of the identified random ten unigenes explored by differential RNA-seq in *S. exigua* larvae after EβF treatments at different doses. **(A)** The relative expression of six upregulated unigenes in *S. exigua* after EβF treatments at different doses. **(B)** The relative expression of four downregulated unigenes in *S. exigua* after EβF treatments at different doses. CK (blank control) and SC (solvent control) were set as controls. The concentrations of EβF treatments were set as 0.16, 0.8, and 4 g/kg; the units of g/kg in all treatment groups mean the mass ratio of EβF/artificial diet. Different lower-case letters indicated a significant difference determined by performing ANOVA, followed by Tukey’s HSD test (*p* < 0.05).

To verify the differential RNA-seq results of *S. exigua* treated by EβF at a dose of 4 g/kg, 10 genes of *S. exigua* were selected to be performing RT-qPCR based on the annotation information, including six upregulated and four downregulated genes. Among these ten genes, four important juvenile hormone-related genes were selected and the other six were related to insect cuticle protein, fatty acid metabolism, key enzymes of detoxification metabolism, etc. The detailed information on the selected 10 unigenes is listed in Supplementary Tables 1–3, (Figures 3A,B). These results not only proved the accuracy of differential RNA-seq data but also showed that EβF disrupted the metabolism of the juvenile hormone of *S. exigua*. Thus, EβF might be used as an antagonist for disrupting the hormone metabolism of *S. exigua*.

### Relative expression of *SeVg* and *SeVgR* in *Spodoptera exigua* treated with (*E*)-β-farnesene at different concentrations

**FIGURE 4:**
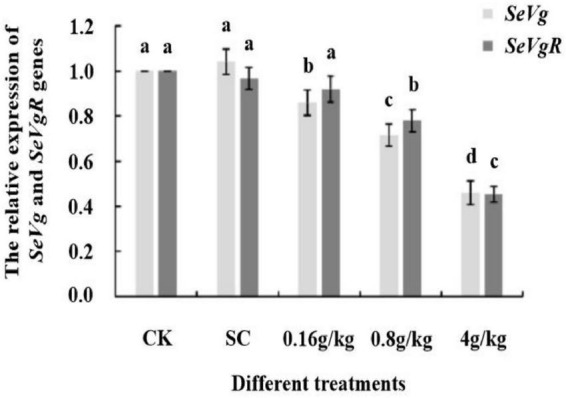
The relative expression of *SeVg* and *SeVgR* genes in female adults of *S. exigua* after EβF treatments at different doses. CK (blank control) and SC (solvent control) were set as controls. The concentrations of EβF treatments were set as 0.16, 0.8, and 4 g/kg; the units of g/kg in all treatment groups means the mass ratio of EβF/artificial diet. Different lower-case letters indicated a significant difference determined by performing ANOVA, followed by Tukey’s HSD test (*p* < 0.05).

The expression levels of *SeVg* decreased significantly with the increase in the concentration of EβF from 0 g/kg (CK and SC controls) to 4 g/kg (*p* < 0.05; Figure 4). After treatment with 4 g/kg of EβF, the expression of *SeVg* was reduced by about 50% compared to its expression in the control groups. After treatment with 4 g/kg of EβF, the expression of *SeVgR* showed a similar change as that of *SeVg*; thus, indicating that EβF affected the formation and accumulation of vitellogenin. Additionally, *SeVgR* expression decreased significantly after treatment with 0.8 and 4 g/kg of EβF compared to its expression in the controls (*p* < 0.05; Figure 4). However, the expression of *SeVgR* showed non-significant change after treatment with 0.16 g/kg of EβF (*p* > 0.05; Figure 4). The expression of *SeVgR* after treatment with 0.8 g/kg of EβF decreased by nearly 50% compared to its expression in the controls.

### Relative expression of the *SeVg and SeVgR* proteins in *Spodoptera exigua* after treatment with different concentrations of (*E*)-β-farnesene

**FIGURE 5:**
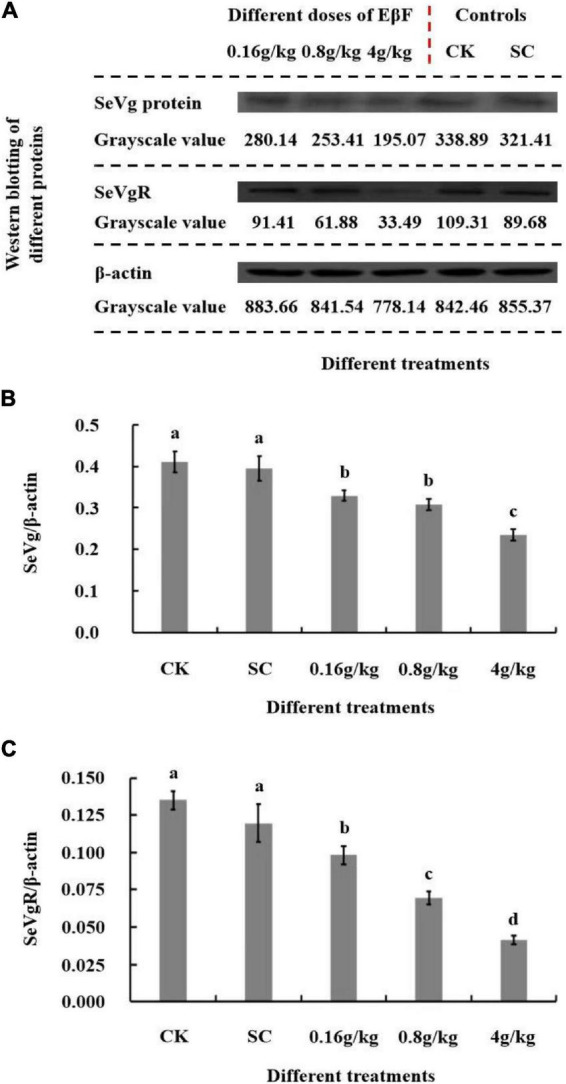
The Western blot assay showed the relative expressions of SeVg and SeVgR proteins in female adults of *S. exigua* after EβF treatments at different doses. **(A)** The Western blot pictures of SeVg and SeVgR proteins in female adults of *S. exigua* after EβF treatments at different doses. **(B,C)** The relative expressions of SeVg and SeVgR protein expressions by calculating the grayscale value ratios of SeVg/β-actin and SeVgR/β-actin with different treatments. CK (blank control) and SC (solvent control) were set as controls. The concentrations of EβF treatments were set as 0.16, 0.8, and 4 g/kg; the units of g/kg in all treatment groups mean the mass ratio of EβF/artificial diet. Different lower-case letters indicated a significant difference determined by performing ANOVA, followed by Tukey’s HSD test (*p* < 0.05).

The results of the Western blot analysis showed that the level of expression of the SeVg and SeVgR proteins was significantly reduced after treatment with 0.16, 0.8, and 4 g/kg of EβF compared to their expression in the two control groups (*p* < 0.05; Figure 5). The reduction in per female fecundity of *S. exigua* after treatment with EβF might be due to a decrease in the expression of *SeVg* and *SeVgR*, which reduced the intrinsic growth rate and limited the population size of *S. exigua.*

### The linear correlation analysis

The linear correlation analysis indicated that the expression of *SeVg* and *SeVgR* was significantly positively correlated with per female fecundity (*p* = 0.0045 and *p* = 0.0001, respectively; Table 3), hatching rate of eggs (%) in *S. exigua* (*p* = 0.067 and *p* = 0.0003, respectively; Table 3) and the intrinsic growth rate (*p* = 0.0045 and *p* = 0.0001, respectively; Table 3). Furthermore, a significant correlation between the hatching rate of eggs (%) and per female fecundity was also found (*p* = 0.0006, Table 3). In addition, the intrinsic rate of increase was significantly positively correlated with per female fecundity (*p* = 0.0001; Table 3) and hatching rate of eggs (%) in *S. exigua* (*p* = 0.0003; Table 3). Finally, the expression level of *SeVg* was positively and significantly correlated with the expression level of *SeVgR* (*p* = 0.0059, Table 3).

**TABLE 3:** The linear correlations between respective key life parameters and *SeVgR*/*SeVg* expression in *S. exigua* after treatment.

Key life parameters (y)	Expression and other parameters (x)	Linear equation	*R* ^2^	*P*	*F*	*SE*
Per female fecundity (y)	The expression of *SeVg* (x)	y = 614.358x–99.833	0.953	0.0045*	60.215	37.406
	The expression of *SeVgR* (x)	y = 663.605x–145.490	0.999	0.0001*	2507.210	5.936
Hatching rate of eggs (%) (y)	The expression of *SeVg* (x)	y = 17.216x + 50.663	0.938	0.0067*	45.465	1.206
	The expression of *SeVgR* (x)	y = 18.671x + 49.322	0.992	0.0003*	356.107	0.443
Intrinsic rate of increase (y)	The expression of *SeVg* (x)	y = 0.129x + 0.025	0.952	0.0045*	59.663	0.008
	The expression of *SeVgR* (x)	y = 0.139x + 0.016	0.998	0.0001*	2300.883	0.001
Per female fecundity (y)	Hatching rate of eggs (%) (x)	y = 35.184x–1875.302	0.987	0.0006*	228.106	19.564
	Intrinsic rate of increase (x)	y = 4773.543x–219.2	0.999	0.0001*	2897.987	5.522
Hatching rate of eggs (%) (y)	Intrinsic rate of increase (x)	y = 134.340x + 47.244	0.992	0.0003*	385.646	0.426
The expression of SeVg (y)	The expression of *SeVgR* (x)	y = 1.024x–0.028	0.943	0.0059*	48.94	0.065

Data are mean ± standard error; different lower-case letters indicate a significant difference obtained by ANOVA, followed by Tukey’s HSD test (“*” means p < 0.05).

## Discussion

Nowadays, exploring natural or synthetic chemicals to kill pests efficiently with the least harm to human or non-target organisms is the focus of studies on pest control (Huang et al., 2021; Tudi et al., 2021). The development of transgenic technology may not only effectively reduce the use of chemical pesticides but also improve the quantity and quality of crops (Kos et al., 2009; Babar et al., 2020). Many transgenic plants are insect resistant, and the most notable one is Bt cotton. Insecticidal toxins from *Bacillus thuringiensis* (Bt) can provide resistance to the main cotton pest bollworm *Helicoverpa armigera* (Wu et al., 2008). Additionally, rice containing the Bt gene can produce the protein Cry1Ac, which can effectively control *C. suppressalis* larvae (Han et al., 2014). However, transgenic technology relies heavily on the discovery of new insect-resistance genes. To achieve a better and wider anti-insect effect, effective insect resistance genes in plants have become an important part of developing transgenic technologies.

The insect resistance gene *tps*46 was identified in rice (Sun et al., 2017). By overexpressing *tps*46, eight kinds of metabolites were produced, and the dose ratio of these eight metabolites to the total metabolites was close to 1:10^4^ (Sun et al., 2017). Therefore, standard samples of the secondary metabolites were added to the artificial diet at a ratio of 1:10^4^ to determine the effects of *tps*46 metabolites on pests, such as *C. suppressalis* (Yang et al., 2022) and *S. exigua* (pre-experiment of this study). Our results also indicated that 0.8 g/kg of EβF has a strong inhibitory effect on *C. suppressalis* larvae, at which concentration of EβF treatment was also reported that the larvae of *C. suppressalis* were almost all died and were unable to complete their life cycle (Yang et al., 2022). However, at the same concentration of EβF, *S. exigua* completed its life cycle. Furthermore, 0.8 and 4 g/kg of EβF significantly reduced the survival of *S. exigua* larvae (*p* < 0.05), per female fecundity (*p* < 0.05), and the intrinsic rate of increase value (*p* < 0.05). Thus, the growth of the population of *S. exigua* was strongly inhibited. These results suggested that genes, such as *tps*46 and *tps*10, which regulate EβF production, might be used as potential resistance genes for pest control (Sun et al., 2017).

After EβF treatment, *S. exigua* larvae showed abnormal changes, including high mortality of the first and second instar larvae and abnormal pupation of the final mature larvae, compared to the control. Because EβF and juvenile hormone are terpenes and have a similar structure (Noriega, 2014; Li et al., 2021), EβF might affect the normal metabolism of juvenile hormone and disrupt the balance between juvenile hormone and ecdysone, which is essential for normal growth, metamorphosis, and reproduction, eventually causing the death of the *S. exigua* larvae (Xu et al., 2021). EβF impairs the development and survival of *C. suppressalis* larvae by disrupting the normal metabolism of juvenile hormone and interfering with the normal hormone balance (Yang et al., 2022). Based on the differential RNA-seq technique, the *S. exigua* juvenile hormone epoxide hydrolase-related genes (Unigene026066, Unigene025818, and Unigene025841) and the hormone ester-like enzyme-related genes (Unigene024849 and Unigene021363) were found to be significantly upregulated (*p* < 0.05). Upregulation of these genes increased the degradation of juvenile hormone and disrupted its metabolism (Kamita et al., 2003; El-Sheikh et al., 2016). The details of these genes are presented in Supplementary Table 1. Juvenile hormone is the main gonadotropin of insects. It maintains the larval state and promotes the development of adult ovaries, which is required for reproduction (Gilbert et al., 2000). This could be one of the reasons why some larvae developed late and eventually died, and some successfully developed into adults but per female fecundity was significantly decreased (*p* < 0.05).

The data from the life table showed that per female fecundity and the intrinsic growth rate of *S. exigua* after treatment with 0.8 and 4 g/kg of EβF decreased significantly (*p* < 0.05). These results were similar to those of a study in which 400 ppm of nerolidol significantly inhibited the spawning of *Spodoptera littoralis* (Ghoneim, 2020), thus indicating that plant secondary metabolites can inhibit the oviposition of pests effectively. Generally known, vitellogenesis is essential for insect reproduction and involves the synthesis of vitellogenin (Vg) and the accumulation of yolk (Xu et al., 2021). In *S. exigua*, *Vg* (*SeVg*) and *Vg* receptor (*SeVgR*) are the key genes associated with yolk synthesis and accumulation. Vg is the main nutrient for egg development in pests, and the vitellogenin receptor plays a key role in the uptake of *SeVg* by oocytes and transport to the ovary (Zhao et al., 2018; Thomas et al., 2020; Hafeez et al., 2021; Wang et al., 2021).

Our results also showed that with an increase in the dose of EβF, the level of expression of both *SeVg* and *SeVgR* decreased significantly (*p* < 0.05). The results of the linear correlation analysis showed that the expression levels of *SeVg* and *SeVgR* after treatments with EβF were all significantly related to per female fecundity, the intrinsic growth rate, and the hatching rate of eggs (%) (*p* < 0.05). All the above findings suggested that EβF should first disturb the normal metabolism of juvenile hormone and break down the balance of juvenile hormone/ecdysone in larvae of *S. exigua*, and then, it inhibited the expressions of *SeVg*/*SeVgR* genes and proteins in female adults of *S. exigua*. Finally, EβF inhibited the population growth of *S. exigua* by increasing larval mortality, reducing per female fecundity, and inhibiting the intrinsic growth rate. Therefore, our results in this study indicated that the main plant secondary metabolite, EβF, might be used as a new environmental-friendly insecticidal compound to control the spread of *S. exigua* in crops.

## Data availability statement

The datasets presented in this study can be found in online repositories. The names of the repository/repositories and accession number(s) can be found below: National Center for Biotechnology Information (NCBI) BioProject database under accession number: PRJNA764052 (https://www.ncbi.nlm.nih.gov/bioproject/PRJNA764052).

## Author contributions

YS, H-YD, LY, and Y-JZ conceived and designed the experiments. YS, YL, BJ, WZ, S-MT, and Y-JZ contributed to manuscript writing. YS, YL, WZ, S-MT, BJ, X-TX, Y-XS, LY, and H-YD conducted the experiment. YS, YL, WZ, and S-MT contributed to the data analysis. All authors contributed to the article and approved the submitted version.

## Footnotes

1
https://github.com/TransDecoder/TransDecoder

## Supplementary material

Supplementary Table 1 The annotation of selected ten unigenes.

Supplementary Table 2 1,345 differentially expressed genes between EβF vs. CK.

Supplementary Table 3 1,345 differentially expressed genes between EβF vs. SC.
